# A review of the subfamily Acaenitinae Förster, 1869 (Hymenoptera, Ichneumonidae) from Ukrainian Carpathians

**DOI:** 10.3897/BDJ.1.e1008

**Published:** 2013-12-10

**Authors:** Alexander Varga

**Affiliations:** † I.I. Schmalhausen Institute of Zoology of National Academy of Sciences of Ukraine, Kiev, Ukraine

**Keywords:** Parasitoids, Ichneumonidae, Acaenitinae, Ukraine, new records, new synonymy

## Abstract

Ichneumonid wasps of the subfamily Acaenitinae Förster, 1869 are reviewed for the first time from the Ukrainian Carpathians. Two species, *Coleocentrus exareolatus* Kriechbaumer, 1894 and *Coleocentrus heteropus* Thomson, 1894 are new records for Ukraine. *Arotes annulicornis* Kriechbaumer, 1894 is considered to be a junior synonym of *Arotes albicinctus* Gravenhorst, 1829 (**syn. nov.**). A key to species of *Coleocentrus* of the Carpathians is provided.

## Introduction

The subfamily Acaenitinae Förster, 1869 worldwide includes about 344 species placed in 27 genera, 8 genera and 42 species of which are found in the Western Palaearctic (Yu et al. 2012).

Rather little is known of the biology of acaenitines. Some Acaenitini are koinobiont endoparasitoids (Shaw and Wahl 1989, Zwakhals 1989). These authors observed that *Acaenitus dubitator* (Panzer, 1800) develops as a koinobiont endoparasitoid of an endophytic curculionid. The female wasp oviposits into first and second instar host larvae and parasitization appeared to retard host development. Three ichneumonid larval instars were discerned and the final instar apparently killed its host about the time unparasitized weevil larvae were pupating. The acaenitine larva then spun a tough parchment-like cylindrical cocon within the host’s pupation chamber (Shaw and Wahl 1989).

The Ukrainian Carpathians are part of the Eastern Carpathians mountain chain and rise in the west of Ukraine within the Lviv, Ivano-Frankivsk, Transcarpathian and Chernivtsi regions. The Carpathians of Ukraine extend from north-west to south-east as a stripe 270 km in length and 100-110 km in width. The Ukrainian Carpathians have an average height of 1000 m and the highest point is 2061 m a.s.l., with relatively soft rocks. Such high-altitude zones as foothill oak forest zone (300–400 m a.s.l.), beech forest zone (400–1300 m a.s.l.), coniferous boreal forest zone (900–1600 m a.s.l.), subalpine and alpine zone (1400–2061 m a.s.l.) can be recognised.

The Acaenitinae fauna of Ukraine is poorly studied. Up to now, there are only 10 recorded species (Besser 1835, Meyer 1934, Kasparyan 1981), but there are no data about distribution of the acaenitins in the Ukrainian Carpathians, while the Romanian fauna is more species-rich and comprises 22 species (Constantineanu and Pisica 1977), some of which can possibly also be distributed in the Ukrainian part of the Carpathians.

## Materials and methods

This study is mainly based on specimens collected by standard sweep netting in various locations in the Ukrainian Carpathians in 2009-2013. The material deposited in the collection of the Vasyl Stefanyk Precarpathian National University in Ivano-Frankivsk was also studied. The *ovipositor‑hind tibia index* (in text OTI), which is the length of the ovipositor projecting beyond the apex of the metasoma divided by the length of the hind tibia, is used. Terminology was Townes (1969) followed. Some specimens of European species of acaenitines were examined from the following collections:

HNHM: Hungarian Natural History Museum, Hungary;

ZMLU: Lunds Universitet, Zoologiska Institutionen, Sweden;

ZIN: Zoological Institute, Russian Academy of Sciences, Russia.

## Taxon treatments

### Arotes

Gravenhorst, 1829

Arotes Arotes albicinctus

#### Diagnosis

This genus is characterized by the combination of the following characters: clypeus transverse and basally flat, with transverse ridge, supra-antennal area with crest between antennal sockets, propodeum with well definded carinae, claws of all tarsi with appressed acute tooth, fore wing with areolet absent, intercubitus distal to vein 2m-cu, first metasomal tergite with white long setae on lateral and ventral parts.

### Arotes albicinctus

Gravenhorst, 1829

Arotes annulicornis Kriechbaumer, 1894, syn. nov.

#### Materials

- **Type status:** Other material. **Occurrence:** recordedBy: Varga; individualCount: Varga; sex: female; **Location:** country: Ukraine; stateProvince: Transcarpathian Region, Rakhiv District; verbatimLocality: 4 km NE of Kvasy; verbatimElevation: 1000 m; verbatimLatitude: 48° 10' 19.08" N; verbatimLongitude: 24° 18' 09.16" E; **Event:** eventDate: 15 June 2012
- **Type status:** Other material. **Occurrence:** recordedBy: Varga; sex: 1 male, 2 females; **Location:** country: Ukraine; stateProvince: Transcarpathian Region, Rakhiv District; verbatimLocality: 4 km NE of Kvasy; verbatimElevation: 1000 m; verbatimLatitude: 48° 10' 19.08" N; verbatimLongitude: 24° 18' 09.16" E; **Event:** eventDate: 24 June 2013

#### Description

##### General features

Fore wing 13 mm long. Nervellus broken near the middle. Mandible with lower tooth longer than upper tooth. Flagellum with 37 segments. Head polished, face and partly clypeus with median longitudinal wrinkles. In dorsal view temples parallel behind eyes. Notauli strong. Mesopleuron, metapleuron, scutellum, mid and hind coxa and hind femur densely and clearly punctate. Metasoma polished, without well defined punctation. OTI 2.3. Hind femur robust.

*Female*. Head, mesosoma and metasoma black. Coloration of first and second tergites varies (see "Taxon discussion") (Fig. 1a, b, c). Clypeus and mandibles black. Flagellum black with white ring, scape and pedicel black. Legs black: all coxae, trochanters and trochantelli almost black, fore and mid tibiae and tarsi ventrally, apex of fore and mid femora, base of hind femur and tibia and tarsomeres 2–5 of hind tarsus white.

*Male*. Coloration as in female, but differs by face, tegula entirely andpedicel partly yellow. Flagellum yellow ventrally, without white ring. First and second tergites with wide apical light stripes (Fig. 1d).

#### Biology

##### Hosts

*Plagionotus* (Sheng et al. 2002), *Plagionotus arcuatus* (Constantineanu and Pisica 1977) (Cerambycidae).

#### Distribution

Albania (Kolarov 1992), Austria (Kazmierczak 1991), Azerbaijan (Meyer 1934), Belarus (Tereshkin 1989), Bulgaria (Kolarov 1997), China (Sheng and Sun 2007), former Czechoslovakia (Sedivy 1989), Finland (Hellén 1940), France (Aubert 1968), Georgia (Meyer 1934), Germany (Horstmann 2001), Hungary (Aubert 1969), Iran (Masnadi-Yazdinejad et al. 2010), Italy (Masi 1948), Korea (Uchida 1955), Netherlands (Zwakhals 1989), Poland (Kazmierczak 2004), Portugal (Blanchard 1840), Romania (Constantineanu and Pisica 1977), Armenia, Russia (Altay Terr., Chita Reg., Irkutsk Reg., Khabarovsk Terr., Primor'ye Terr.) (Kasparyan and Khalaim 2007), Spain (Anento and Selfa 1996), Turkey (Kolarov 1995), Ukraine (Besser 1835), United Kingdom (Kloet and Hincks 1945).

#### Notes

The another European species, *Arotes ustulatus* Kriechbaumer, 1894, differs from *Arotes albicinctus* Gravenhorst, 1829 in coloration of legs (which are red except coxae) and in the possession of a fuscous spot on the apex of the fore wing.

#### Taxon discussion

The main distinguishing characters, between *Arotes albicinctus* Gravenhorst, 1829 and *Arotes annulicornis* Kriechbaumer, 1894, given by various authors are the coloration of the first and second tergites of metasoma, which are entirely black in *Arotes annulicornis* Kriechbaumer, 1894 and light-coloured posteriorly in *Arotes albicinctus* Gravenhorst, 1829, and pterostigma, which is reddish centrally in *Arotes annulicornis* Kriechbaumer, 1894 and entirely fuscous in *Arotes albicinctus* Gravenhorst, 1829. Kolarov (1997) and Constantineanu and Pisica (1977) gave another character, the length of ovipositor sheaths, which are slightly shorter than body in *Arotes annulicornis* Kriechbaumer, 1894 and as long as the body in *Arotes albicinctus* Gravenhorst, 1829.

My examination of the holotype of *Arotes annulicornis* Kriechbaumer, 1894, which is deposited at HNHM, demonstrated that the first and the second tergites have light-coloured (though very weak) posterior margins. The three of metioned above females (collected in the same locality) have first and second tergites varies from entirely black to white-striped. The coloration of the pterostigma is also varies in the studied specimens: from yellowish-brown centrally with fuscous margins to entirely fuscous. The ovipositorial sheaths are as long as the body in the specimens with black tergites, so no evident differences between the two species, *Arotes annulicornis* Kriechbaumer, 1894 and *Arotes albicinctus* Gravenhorst, 1829, may be found. *Arotes annulicornis* Kriechbaumer, 1894 is therefore a junior synonym (syn. nov.).

### Coleocentrus

Gravenhorst, 1829

Coleocentrus Ichneumon excitator

#### Diagnosis

This genus is characterized by the combination of the following characters: clypeus transverse and basally flat, apex with median tubercle, supra-antennal area without crest between antennal sockets, epicnemial carina absent, propodeum with carinae varying from complete to absent (usually only dorsal longitudinal carinae), claws of fore and mid tarsi simple, fore wing with areolet present (petiolate triangular) or absent; if absent (*Coleocentrus exareolatus* Kriechbaumer, 1894), then intercubitus basal to vein 2m-cu, tergites 2–3 of metasoma with basolateral grooves, male parameres with ventral emargination.

### Coleocentrus exareolatus

Kriechbaumer, 1894

#### Materials

- **Type status:** Other material. **Occurrence:** recordedBy: Varga; sex: 2 males; **Location:** country: Ukraine; stateProvince: Transcarpathian Region, Rakhiv District; verbatimLocality: 4 km NE of Kvasy; verbatimElevation: 1000 m; verbatimLatitude: 48° 10' 19.08" N; verbatimLongitude: 24° 18' 09.16" E; **Event:** eventDate: 27 May 2011
- **Type status:** Other material. **Occurrence:** sex: female; **Record Level:** institutionCode: ZIN

#### Description

##### General features

Fore wing 17 mm long (in female) and 12-14 mm long (in male), areolet absent. Nervellus broken at upper 0.25. Mandible with equal teeth or lower tooth slighty longer than upper tooth. Flagellum with 37-38 segments. Head polished. In dorsal view temples parallel to narrowed behind eyes. Propodeum with weak apical carina. Metasoma matt, without well defined punctation. OTI 2.7.

*Female*. Head and mesosoma black. Clypeus basally black, apically brownish. Mandibles black. Flagellum brownish. Pterostigma yellowish. Tegula yellow. Legs generally red, fore and mid tibia and tarsus yellowish-red, hind tibia and tarsus fuscous. Metasoma black with narrow apical white bands on tergites.

*Male*. Head and mesosoma black. Face and clypeus black. Mandibles black. Flagellum black. Scape and pedicel yellow dorsally. Pterostigma yellowish. Tegula yellow. Legs: hind coxa red, fore and mid coxae, trochanters and trochantelli, tibiae and fore tarsus yellow, fore and mid femora, hind trochanter and trochantellus yellowish-red, hind tibia and tarsus fuscous. Metasoma black with narrow apical white bands on tergites.

#### Biology

##### Hosts

Unknown.

#### Distribution

Belarus (Tereshkin 1987), Bulgaria (Kolarov 1997), Germany (Horstmann 2001), Hungary (Kiss von Zilah 1924), Latvia (Ozols 1958), Poland (Kazmierczak 2004), Romania (Constantineanu and Pisica 1977), Russia (Kamchatka Reg., Sankt Petersburg, Sakhalin Reg., Khabarovsk Terr., Primor'ye Terr.) (Meyer 1934, Kasparyan and Khalaim 2007), new for Ukraine.

#### Notes

The female of another European species with fore wing without areolet, *Coleocentrus soldanskii* Bischoff, 1915, has black coxae and two yellow spots on the lower part of face.

### Coleocentrus excitator

(Poda, 1761)

#### Materials

- **Type status:** Other material. **Occurrence:** recordedBy: Varga; sex: male; **Location:** country: Ukraine; stateProvince: Ivano-Frankivsk Region, Bogorodchany District, Mochary; verbatimLocality: 5 km NE of Bogorodchany; verbatimElevation: 300-350 m; verbatimLatitude: 48° 50' 51.17" N; verbatimLongitude: 24° 35' 26.91" E; **Event:** eventDate: 5 May 2012
- **Type status:** Other material. **Occurrence:** recordedBy: Varga; sex: male; **Location:** country: Ukraine; stateProvince: Ivano-Frankivsk Region, Bogorodchany District, Mochary; verbatimLocality: 5 km NE of Bogorodchany,; verbatimElevation: 300-350 m; verbatimLatitude: 48° 50' 51.17" N; verbatimLongitude: 24° 35' 26.91" E; **Event:** eventDate: 6 May 2012
- **Type status:** Other material. **Occurrence:** recordedBy: Varga; sex: 2 females; **Location:** country: Ukraine; stateProvince: Ivano-Frankivsk Region, Bogorodchany District, Mochary; verbatimLocality: 5 km NE of Bogorodchany; verbatimElevation: 300-350 m; verbatimLatitude: 48° 50' 51.17" N; verbatimLongitude: 24° 35' 26.91" E; **Event:** eventDate: 12 May 2012
- **Type status:** Other material. **Occurrence:** recordedBy: Varga; sex: female; **Location:** country: Ukraine; stateProvince: Ivano-Frankivsk Region, Bogorodchany District, Mochary; verbatimLocality: 5 km NE of Bogorodchany; verbatimElevation: 300-350 m; verbatimLatitude: 48° 50' 51.17" N; verbatimLongitude: 24° 35' 26.91" E; **Event:** eventDate: 19 May 2012
- **Type status:** Other material. **Occurrence:** recordedBy: Varga; sex: 1 male, 3 females; **Location:** country: Ukraine; stateProvince: Ivano-Frankivsk Region, Bogorodchany District, Mochary; verbatimLocality: 5 km NE of Bogorodchany; verbatimElevation: 300-350 m; verbatimLatitude: 48° 50 '51.17" N; verbatimLongitude: 24° 35' 26.91" E; **Event:** eventDate: 29 May 2012
- **Type status:** Other material. **Occurrence:** recordedBy: Varga; sex: female; **Location:** country: Ukraine; stateProvince: Ivano-Frankivsk Region, Bogorodchany District, Mochary; verbatimLocality: 5 km NE of Bogorodchany; verbatimElevation: 300-350 m; verbatimLatitude: 48° 50' 51.17" N; verbatimLongitude: 24° 35' 26.91" E; **Event:** eventDate: 30 May 2011
- **Type status:** Other material. **Occurrence:** recordedBy: Varga; sex: 6 females; **Location:** country: Ukraine; stateProvince: Ivano-Frankivsk Region, Bogorodchany District, Mochary; verbatimLocality: 5 km NE of Bogorodchany; verbatimElevation: 300-350 m; verbatimLatitude: 48° 50' 51.17" N; verbatimLongitude: 24° 35' 26.91" E; **Event:** eventDate: 31 May 2012
- **Type status:** Other material. **Occurrence:** recordedBy: Varga; sex: female; **Location:** country: Ukraine; stateProvince: Ivano-Frankivsk Region, Bogorodchany District, Mochary; verbatimLocality: 5 km NE of Bogorodchany; verbatimElevation: 300-350 m; verbatimLatitude: 48° 50' 51.17" N; verbatimLongitude: 24° 35' 26.91" E; **Event:** eventDate: 4 June 2011
- **Type status:** Other material. **Occurrence:** recordedBy: Varga; sex: 5 females; **Location:** country: Ukraine; stateProvince: Ivano-Frankivsk Region, Bogorodchany District, Mochary; verbatimLocality: 5 km NE of Bogorodchany; verbatimElevation: 300-350 m; verbatimLatitude: 48° 50' 51.17" N; verbatimLongitude: 24° 35' 26.91" E; **Event:** eventDate: 10 June 2012
- **Type status:** Other material. **Occurrence:** recordedBy: Varga; sex: female; **Location:** country: Ukraine; stateProvince: Ivano-Frankivsk Region, Bogorodchany District, Mocharyorodchany, 5 May 2012; verbatimLocality: 5 km NE of Bogorodchany; verbatimElevation: 300-350 m; verbatimLatitude: 48° 50' 51.17" N; verbatimLongitude: 24° 35' 26.91" E; **Event:** eventDate: 25 June 2012
- **Type status:** Other material. **Occurrence:** recordedBy: Varga; sex: 2 males, 1 female; **Location:** country: Ukraine; stateProvince: Ivano-Frankivsk Region, Bogorodchany District, Zhbyr; verbatimLocality: 7-8 km SW of Bogorodchany; verbatimElevation: 400 m; verbatimLatitude: 48° 47' 4.92" N; verbatimLongitude: 24° 28' 46.45" E; **Event:** eventDate: 23 May 2012
- **Type status:** Other material. **Occurrence:** recordedBy: Varga; sex: male; **Location:** country: Ukraine; stateProvince: Ivano-Frankivsk Region, Bogorodchany District, Zhbyr; verbatimLocality: 7-8 km SW of Bogorodchany; verbatimElevation: 400 m; verbatimLatitude: 48° 47' 4.92" N; verbatimLongitude: 24° 28' 46.45" E; **Event:** eventDate: 26 May 2012
- **Type status:** Other material. **Occurrence:** recordedBy: Varga; sex: female; **Location:** country: Ukraine; stateProvince: Zhbyr; verbatimLocality: Ivano-Frankivsk Region, Bogorodchany District, Zhbyr; verbatimElevation: 400 m; verbatimLatitude: 48° 47' 4.92" N; verbatimLongitude: 24° 28' 46.45" E; **Event:** eventDate: 24 June 2012
- **Type status:** Other material. **Occurrence:** recordedBy: Varga; sex: 2 females; **Location:** country: Ukraine; stateProvince: Ivano-Frankivsk Region, Bogorodchany District, Dibrova.28"E, 310 m, oak forest, 5 km SW of Bogorodchany, 14 June 2012; verbatimLocality: 5 km SW of Bogorodchany; verbatimElevation: 310 m; verbatimLatitude: 48° 46' 10.35" N; verbatimLongitude: 24° 30' 20.28" E; **Event:** eventDate: 14 June 2012
- **Type status:** Other material. **Occurrence:** recordedBy: Varga; sex: 2 males, 2 females; **Location:** country: Ukraine; stateProvince: Ivano-Frankivsk Region, Bogorodchany District, Gorgany; verbatimLocality: 5 km SW of Stara Guta; verbatimElevation: 1200 m; verbatimLatitude: 48° 36' 42.77" N; verbatimLongitude: 24° 09' 10.69" E; **Event:** eventDate: 8-9 June 2012
- **Type status:** Other material. **Occurrence:** recordedBy: Varga; sex: 2 males; **Location:** country: Ukraine; stateProvince: Ivano-Frankivsk Region, Bogorodchany District, Gorgany; verbatimLocality: 5 km SW of Stara Guta; verbatimElevation: 1200 m; verbatimLatitude: 48° 36' 42.77" N; verbatimLongitude: 24° 09' 10.69" E; **Event:** eventDate: 14 June 2011
- **Type status:** Other material. **Occurrence:** recordedBy: Sirenko; sex: female; **Location:** country: Ukraine; stateProvince: Ivano-Frankivsk Region, Nadvirna District, Gorgany, Elmy; verbatimLocality: 15 km SW of Yaremche; verbatimElevation: 800-900 m; verbatimLatitude: 48° 24’ 39.50” N; **Event:** eventDate: 9 July 2005
- **Type status:** Other material. **Occurrence:** recordedBy: Sirenko; sex: female; **Location:** country: Ukraine; stateProvince: Ivano-Frankivsk Region, Nadvirna District, Gorgany, Elmy; verbatimLocality: 15 km SW of Yaremche; verbatimElevation: 800-900 m; verbatimLatitude: 48° 24’ 39.50” N; verbatimLongitude: 24° 24’ 50.28” E; **Event:** eventDate: 17 July 2009
- **Type status:** Other material. **Occurrence:** sex: female; **Record Level:** institutionCode: ZIN

#### Description

##### General features

Fore wing 18-20 mm long (in female) and 13-14 mm long (in male), areolet present. Nervellus broken at upper 0.25. Mandible with equal teeth or lower tooth slightly longer than upper tooth. Flagellum with 39-40 segments (in female) and 40-44 segments (in male). Head polished, sparsely punctate. In dorsal view temples slighty narrowed to slighty widened behind eyes. Mesopleuron densely rugulo-punctate (in female) or with unclear punctation (in male). Propodeum with weak longitudinal carinae over about 0.6-0.7 of its length (in male) or only with weak traces of dorsal longitudinal carinae (in female). Metasoma polished, without well defined punctation. OTI 2.6–2.8.

*Female*. Head, mesosoma and metasoma black. Face black with two yellow spots. Clypeus basally black, apically sometimes dark-brownish. Mandibles black. Flagellum black. Scape and pedicel reddish dorsally. Pterostigma yellow. Tegula yellow. Legs: hind coxa black, fore and mid coxae black with red apex, all trochanters and trochantelli yellowish-red, all femora, fore and mid tibiae and tarsi red, hind tibia fuscous, tarsomere 1 of hind tarsus partly, tarsomeres 2-5 entirely white. Metasoma black with narrow apical white bands on tergites.

*Male*. Head and mesosoma black. Face yellow with black central vertical stripe. Clypeus basally black, apically brown. Mandibles black. Flagellum dark-brownish. Scape and pedicel yellow dorsally. Pterostigma yellow. Tegula yellow. Legs: hind coxa black, sometimes reddish in apical 0.2, fore and mid coxae yellowish-red with black base, fore and mid femora, hind trochanter and trochantellus yellowish-red, hind femur and tibia red, fore and mid trochanters and trochantelli, tibiae and fore tarsus, tarsomere 1 of hind tarsus partly, tarsomeres 2-5 entirely white. Metasoma black basally and apically, red medially.

#### Biology

##### Hosts

*Acalolepta luxuriosus* (Bates, 1873), *Ergates faber* (Linnaeus, 1761), *Monochamus grandis* (Waterhaus, 1881) (Cerambycidae) (Aubert 1969, Constantineanu and Pisica 1977).

#### Distribution

Trans-Palaearctic species: Belarus (Tereshkin 1987), Belgium (Wesmael 1849), Bulgaria (Kolarov 1997), China (Hong and Sheng 1997), Croatia (Kolarov 2008), Czech Republic (Zeman and Mocek 2006), Finland, France, Netherlands, Poland, Hungary, Sweden, Switzerland (Aubert 1969), Germany (Horstmann 2001), Italy (Scaramozzino 1986), Lithuania (Constantineanu and Jonaitis 1979), Norway (Riedel et al. 2000), Romania (Constantineanu and Pisica 1977), Russia (Astrakhan Reg., Moscow Reg., Omsk Reg., Primor'ye Terr., Sakhalin Reg., Sankt Petersburg, Yaroslavl Reg.) (Meyer 1934, Kasparyan 1981, Kasparyan and Khalaim 2007), Spain (Habermehl 1927), United Kingdom (Shaw 1986), Yugoslavia (Glavendekic and Kolarov 1994), Ukraine (Kasparyan 1981), widespread and common species in Ukrainian Carpathians.

#### Notes

The female of *Coleocentrus croceicornis* (Gravenhorst, 1829) is similar to this species, but has yellow flagellum with black base and entirely reddish hind legs. The male of *Coleocentrus excitator* (Poda, 1761) is similar to the male of *Coleocentrus soleatus* (Gravenhorst, 1829), but the last one has red with black coloration hind trochanters and trochantelli, entirely yellow face and only tarsomeres 3-5 of hind tarsus entirely white.

### Coleocentrus heteropus

Thomson, 1894

#### Materials

- **Type status:** Holotype. **Occurrence:** sex: female; **Record Level:** institutionCode: ZMLU
- **Type status:** Other material. **Occurrence:** recordedBy: Varga; sex: 2 males, 2 females; **Location:** country: Ukraine; stateProvince: Ivano-Frankivsk Region, Bogorodchany District, Zhbyr; verbatimLocality: 7-8 km SW of Bogorodchany,; verbatimElevation: 400 m; verbatimLatitude: 48° 47' 4.92" N; verbatimLongitude: 24° 28' 46.45" E; **Event:** eventDate: 23 May 2012
- **Type status:** Other material. **Occurrence:** recordedBy: Varga; sex: male; **Location:** country: Ukraine; stateProvince: Ivano-Frankivsk Region, Bogorodchany District, Zhbyr; verbatimLocality: 7-8 km SW of Bogorodchany; verbatimElevation: 400 m; verbatimLatitude: 48° 47' 4.92" N; verbatimLongitude: 24° 28' 46.45" E; **Event:** eventDate: 26 May 2012
- **Type status:** Other material. **Occurrence:** recordedBy: Varga; sex: female; **Location:** country: Ukraine; stateProvince: Ivano-Frankivsk Region, Bogorodchany District, Zhbyr; verbatimLocality: 7-8 km SW of Bogorodchany; verbatimElevation: 400 m; verbatimLatitude: 48° 47' 4.92" N; verbatimLongitude: 24° 28' 46.45" E; **Event:** eventDate: 24 June 2012
- **Type status:** Other material. **Occurrence:** sex: female; **Record Level:** institutionCode: ZIN

#### Description

##### General features

Fore wing 14-15 mm long (in female) and 9-13 mm long (in male), areolet present. Nervellus broken at upper 0.25. Mandible with equal teeth. Flagellum with 33 segments (in female) to 38 segments (in male). Head polished, without well definded punctation. In dorsal view temples parallel to slighty widened behind eyes. Mesopleuron densely rugulo-punctate. Propodeum only with weak traces of area apicalis or without carinae. Metasoma matt, without well defined punctation. OTI 2.5.

*Female*. Head, mesosoma and metasoma black. Face black with two small yellow spots or almost black. Clypeus from reddish to dark-brownish. Mandible black. Flagellum almost black. Pterostigma brownish with fuscous margins. Tegula red-yellowish. Legs generally red, all coxae from at least black basally (holotype). to black with only red apex, hind tibia and tarsus fuscous.

*Male*. Head, mesosoma and metasoma black. Face almost yellow. Clypeus red-brownish. Mandibles black. Flagellum black. Scape yellow dorsally. Pterostigma brownish with fuscous margins. Tegula yellow. Legs: all coxae black basally, apically yellowish-red, fore and mid legs except coxae, hind trochanter and trochantellus yellowish-red, hind femur red, hind tibia and tarsus fuscous.

#### Biology

##### Hosts

Unknown.

#### Distribution

Finland (Hellén 1937), Hungary (Kiss von Zilah 1926), Romania (Constantineanu and Pisica 1977), Sweden (Aubert 1969), new for Ukraine.

#### Notes

The female of *Coleocentrus caligatus* Gravenhorst, 1829 is similar to this species, but has entirely red hind coxae and more developed carinae of the propodeum. Male of *Coleocentrus heteropus* Thomson, 1894 is similar to *Coleocentrus croceicornis* (Gravenhorst, 1829), but the last one has reddish pterostigma and yellow flagellum (with dark base).

### Coleocentrus soleatus

(Gravenhorst, 1829)

#### Materials

- **Type status:** Other material. **Occurrence:** recordedBy: Varga; sex: male; **Location:** country: Ukraine; stateProvince: Ivano-Frankivsk Region, Bogorodchany District, Dibrova; verbatimLocality: 5 km SW of Bogorodchany; verbatimElevation: 310 m; verbatimLatitude: 48° 46' 10.35" N; verbatimLongitude: 24° 30' 20.28" E; **Event:** eventDate: 14 May 2011
- **Type status:** Other material. **Occurrence:** recordedBy: Varga; sex: female; **Location:** country: Ukraine; stateProvince: Transcarpathian Region, Rakhiv District, Lazeschyna; verbatimElevation: 900-950 m; verbatimLatitude: 48° 14' 52.47" N; verbatimLongitude: 24° 24' 29.35" E; **Event:** eventDate: June 2010
- **Type status:** Other material. **Occurrence:** sex: male; **Record Level:** institutionCode: ZIN

#### Description

##### General features

Fore wing 11 mm long (in female) and 12 mm long (in male), areolet present. Nervellus broken at upper 0.25. Mandible with equal teeth. Flagellum with 31 segments (in female) and 33 segments (in male). Head polished, without well definded punctation. In dorsal view temples narrowed behind eyes. Mesopleuron weakly (in female) or densely (in male) rugulo-punctate. Propodeum with very weak longitudinal carinae over its entire length (in male) or only with weak traces of dorsal longitudinal carinae (in female). Metasoma mat (in female) or polished (in male), without well defined punctation. OTI 1.8.

*Female*. Head, mesosoma and metasoma generally black. Face almost black. Clypeus black basally, reddish apically. Mandibles reddish basally. Flagellum almost black. Pterostigma brownish with fuscous margins. Tegula reddish-brown. Legs: mid coxae almost black, fore and hind coxae generally red, fore coxa basally, hind coxa apically (0.2) black, all femora, fore and mid tibiae and tarsi red, hind tibia apically and tarsus entirely fuscous, all trochanters and trochantelli red with black coloration. Lateral parts of apical margins of tergites 2-6 red.

*Male*. Head and mesosoma black. Face almost yellow. Clypeus basally black, apically reddish. Mandibles black. Flagellum reddish-brown. Scape yellowish dorsally. Pterostigma yellowish. Tegula reddish-brown. Legs: hind coxa red with only apical 0.2 black, fore and mid coxae, fore trochanter and trochantellus and all femora red, mid and hind trochanters and trochantelli red with black coloration, fore tibia and tarsus, tarsomere 2 of hind tarsus partly, tarsomeres 3-5 entirely white, mid tibia and tarsus yellowish-red, hind tibia red with fuscous apex. Metasoma black basally and apically, red medially.

#### Biology

##### Hosts

Unknown.

#### Distribution

Bulgaria (Kolarov 1997), China (Jilin, Liaoning) (Sheng and Zhang 1999), Germany (Townes et al. 1965), Hungary (Kiss von Zilah 1926), Korea (Uchida 1955), Poland (Hedwig 1937), Romania (Constantineanu and Pisica 1977), Russia (Caucasus), Ukraine (Kasparyan 1981).

#### Notes

Constantineanu and Pisica (1977) recorded *Coleocentrus borcei* Constantineanu, 1929 from Romania, wich is very similar to the female of *Coleocentrus soleatus* (Gravenhorst, 1829), but has a fuscous pterostigma (except base), entirely black trochanters, black clypeus and red lateral parts of apical margins of tergites 3-6.

### Phaenolobus

Förster, 1869

Phaenolobus Ichneumon arator

#### Diagnosis

This genus is characterized by the combination of the following characters: clypeus transverse and basally flat, apex with median tubercle, supra-antennal area with crest between antennal sockets, notauli strong, hing femora very thick claws of fore and mid tarsi with appressed acute tooth near apex, fore wing with areolet absent, intercubitus basal to vein 2m-cu.

### Phaenolobus fulvicornis

(Gravenhorst, 1829)

#### Materials

- **Type status:** Other material. **Occurrence:** recordedBy: Sirenko A.; sex: female; **Location:** country: Ukraine; stateProvince: Ivano-Frankivsk; verbatimElevation: 250–300 m; verbatimLatitude: 48° 55’ 24.48” N; verbatimLongitude: 24° 42’ 40.02” E; **Event:** eventDate: May–June 2001

#### Description

##### General features

*Female*. Fore wing 8 mm long. Nervellus broken at upper third. Mandible with upper tooth a little longer than lower tooth. Flagellum with 23 segments. Head strongly rugulo-punctate. In dorsal view temples narrowed behind eyes. Malar space with subocular groove. Mesopleuron polished, densely and clearly punctate. Head and mesosoma black. Clypeus and mandibles black. Flagellum red-brown, scape and pedicel black. Pterostigma fuscous. Legs: all coxae black, trochanters red and trochantelli, hind femur and basal half of first tergite black, hind tibia and tarsus fuscous, fore and mid femora, tibiae, tarsi and metasoma red.

#### Biology

##### Hosts

*Phytoecia cephalotes* Küster, 1846, *Phytoecia coerulescens* (Scopoli, 1763) (Cerambycidae) (Scaramozzino 1986).

#### Distribution

Albania (Kolarov and Andoni 1995), Algeria, Morocco, Israel, Italy, Portugal, Yugoslavia (Aubert 1969), Belarus (Sawoniewicz 2001), Bulgaria, Croatia, Serbia & Montenegro, Turkey (Kolarov 1995, Kolarov 1997, Kolarov 2008), Georgia (Djanelidze 1966), Germany (Horstmann 2001), Hungary (Kiss von Zilah 1926), Iran (Masnadi-Yazdinejad et al. 2010), Russia (Caucasus, Ryazan Reg.) (Kasparyan 1981), Latvia (Ozols 1958), Lithuania (Constantineanu and Jonaitis 1979), Netherlands (Zwakhals 1989), Poland (Hedwig 1937), Romania (Constantineanu and Pisica 1977), Spain (Mazon et al. 2011), Switzerland (Bauer 2002), Ukraine (Kasparyan 1981).

#### Notes

There are another three species of this genus recorded so far from Ukraine, including *Phaenolobus terebrator* (Scopoli, 1763) with black metasoma and red hind femora, *Phaenolobus nigripennis* (Gravenhorst, 1829) with only tergites 2-4 partly red, *Phaenolobus saltans* (Gravenhorst, 1829) which has prepectal carina long, almost reaching subtegular ridge. Constantineanu and Pisica (1977) additionally recorded another 3 new species, described from Romania, *Phaenolobus areolator* (Constantineanu & Constantineanu, 1968) having the entirely black flagellum and ovipositor longer than hind tibia, *Phaenolobus atrator* (Constantineanu and Pisica, 1977), having the black metasoma and *Phaenolobus mucronatus* (Constantineanu & Constantineanu, 1968) having only tergites 2-4 partly red. But the last two species have also the second metasomal tergite with 2 oblique grooves on each side and that scaracter distinguishes these species from similar *Phaenolobus terebrator* (Scopoli, 1763) and *Phaenolobus nigripennis* (Gravenhorst, 1829) with the same coloration of metasoma respectively (Kolarov and Gürbüz 2010).

## Identification Keys

### Key to species of *Coleocentrus* in Carpathians

**Table:** 

1	Areolet absent. Hind coxa red. Face of both sexes entirely black (Fig. 2a)	***Coleocentrus exareolatus* Kriechbaumer, 1894**
–	Areolet present. Hind coxa red to black. At least face of males partly yellow	2
2	Females	3
–	Males	8
3	Hind trochanter and trochantellus red or yellowish-red	4
–	Hind trochanter and trochantellus red with black coloration (Fig. 2b)	7
4	Apical tarsomeres of hind tarsus white. Hind coxa black. Hind trochanter and trochantellus yellowish-red (Fig. 2d)	***Coleocentrus excitator* (Poda, 1761)**
–	Hind tarsus reddish to fuscous. Hind coxa red to partly black. Hind trochanter and trochantellus red	5
5	Hind tarsus reddish. Hind coxa red. Flagellum yellowish with black base	***Coleocentrus croceicornis* (Gravenhorst, 1829)**
–	Hind tarsus at least apically fuscous. Hind coxa red to partly black. Flagellum black	6
6	Hind tarsus fuscous apically. Hind coxa red. Propodeum with well defined longitudinal carinae and apical transverse carina	***Coleocentrus caligatus* Gravenhorst, 1829**
–	Hind tarsus entirely fuscous. Hind coxa at least black basally (Fig. 2f)	***Coleocentrus heteropus* Thomson, 1894**
7	All trochanters black. Pterostigma fuscous (except base). Clypeus black. Lateral parts of apical margins of tergites 3-6 red	***Coleocentrus borcei* Constantineanu, 1929**
–	At least fore trochanter partly red. Pterostigma reddish centrally. Clypeus apically red. Lateral parts of apical margins of tergites 2-6 red	***Coleocentrus soleatus* (Gravenhorst, 1829)**
8	Face black, with only inner margins of eyes yellow. Pterostigma reddish. Flagellum yellowish	***Coleocentrus croceicornis* (Gravenhorst, 1829)**
–	Face yellow, at most with nurrow central vertical black stripe. Pterostigma yellowish or fuscous. Flagellum reddish-brown to black	9
9	Hind tarsus fuscous at least apically. Metasoma entirely black	10
–	Apical tarsomeres of hind tarsus white. Metasoma red centrally	11
10	Hind coxa black basally. Hind tibia and tarsus entirely fuscous	***Coleocentrus heteropus* Thomson, 1894**
–	Hind coxa black apically. Hind tibia and tarsus red, fuscous apically	***Coleocentrus caligatus* Gravenhorst, 1829**
11	Hind trochanter and trochantellus yellowish-red. Hind coxa black at least in basal 0.8. Face yellow with black central vertical stripe (Fig. 2c)	***Coleocentrus excitator* (Poda, 1761)**
–	Hind trochanter and trochantellus red with black coloration. Hind coxa black at most in apical 0.2. Face entirely yellow (Fig. 2e)	***Coleocentrus soleatus* (Gravenhorst, ​1829)**

## Supplementary Material

XML Treatment for Arotes

XML Treatment for Arotes albicinctus

XML Treatment for Coleocentrus

XML Treatment for Coleocentrus exareolatus

XML Treatment for Coleocentrus excitator

XML Treatment for Coleocentrus heteropus

XML Treatment for Coleocentrus soleatus

XML Treatment for Phaenolobus

XML Treatment for Phaenolobus fulvicornis

## Figures and Tables

**Figure 1a.**
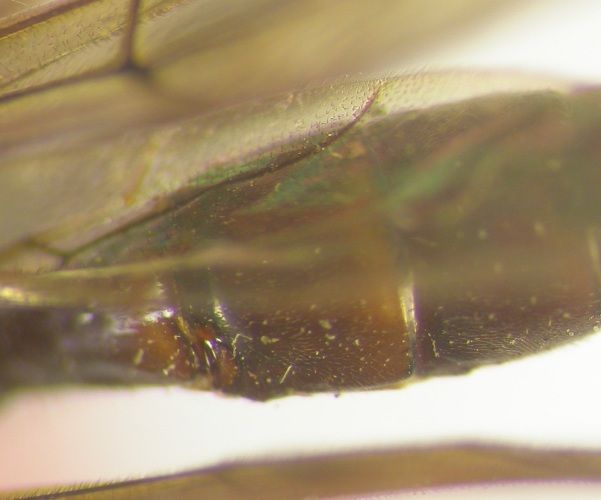
*Arotes annulicornis* Kriechbaumer, 1894, female (holotype).

**Figure 1b.**
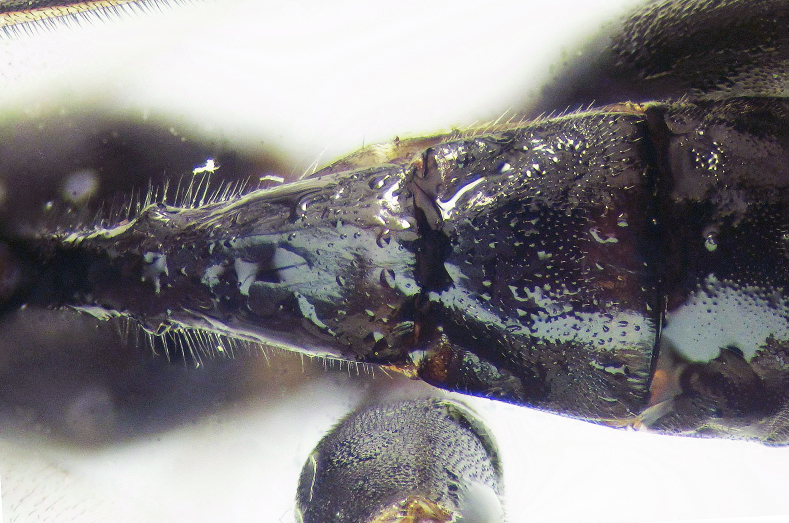
Female 1, collected in Carpathians.

**Figure 1c.**
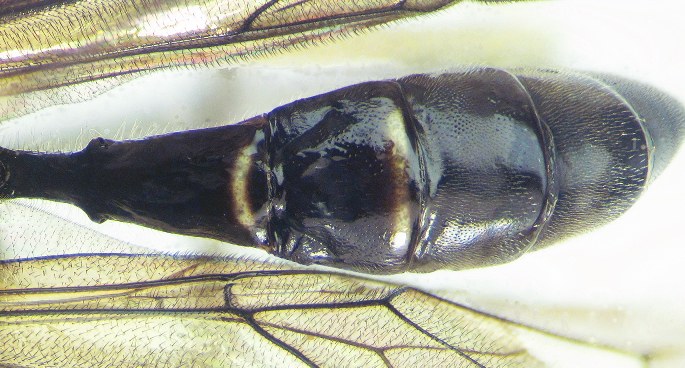
Female 2, collected in Carpathians.

**Figure 1d.**
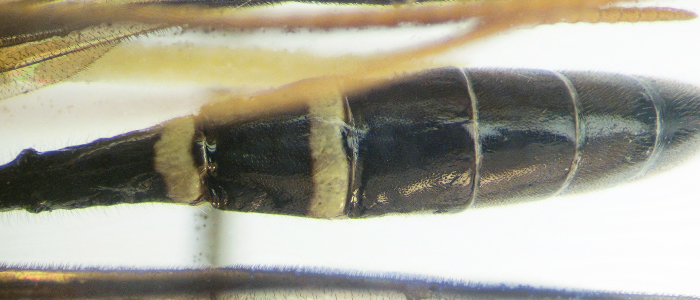
Male, collected in Carpathians.

**Figure 2a.**
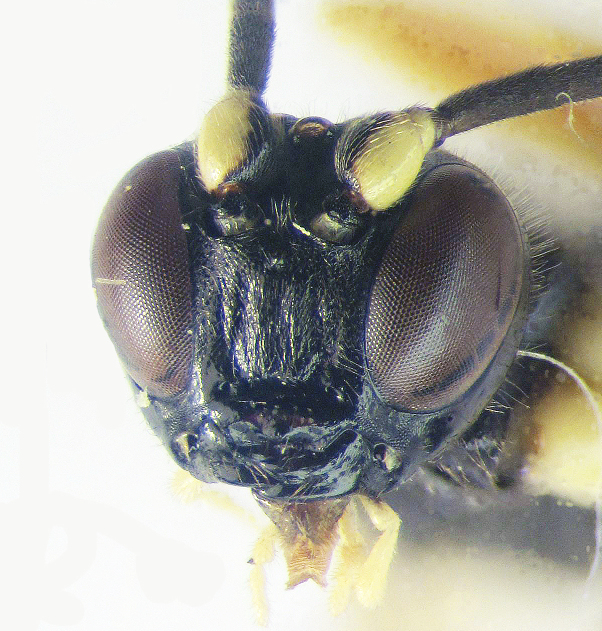
*Coleocentrus exareolatus* Kriechbaumer, 1894, male, face.

**Figure 2b.**
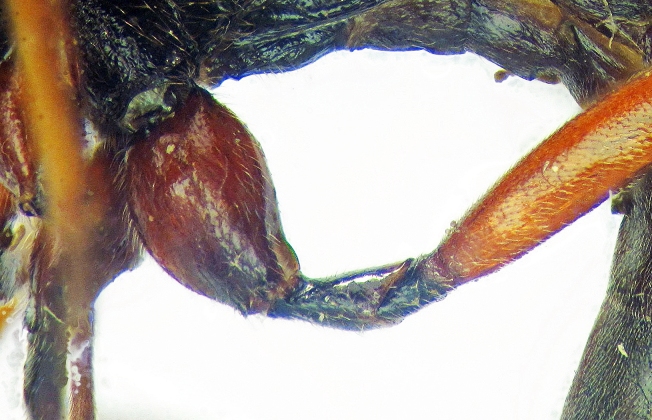
*Coleocentrus soleatus* (Gravenhorst, 1829), female, hind coxa, trochanter and trochantellus.

**Figure 2c.**
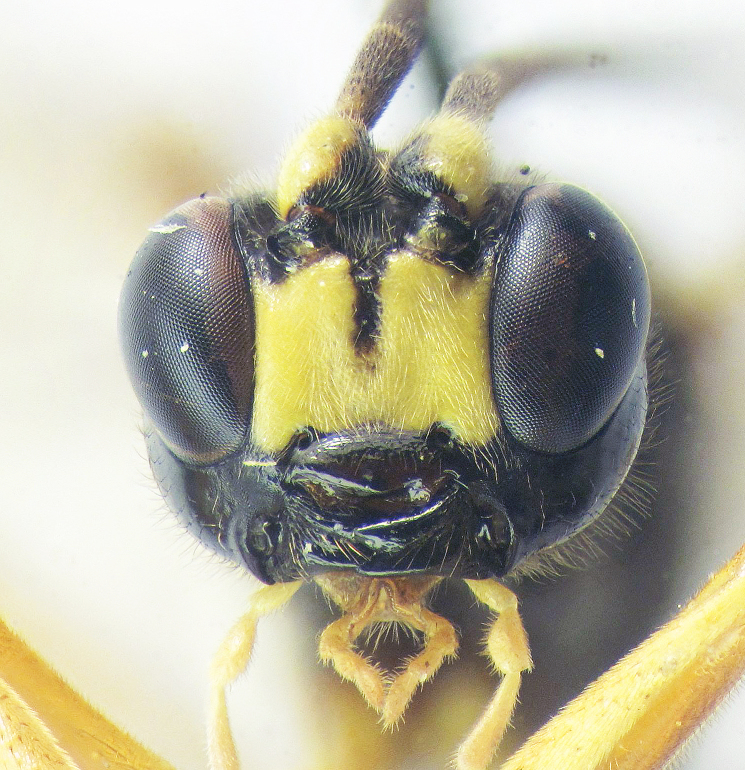
*Coleocentrus excitator* (Poda, 1761), male, face.

**Figure 2d.**
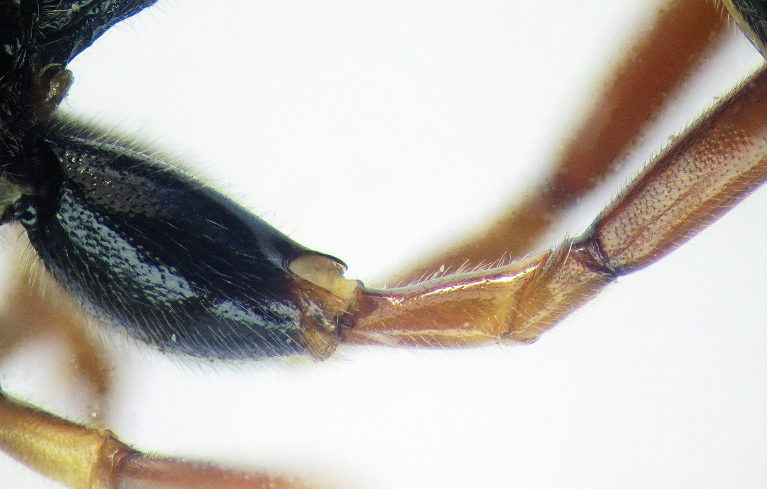
*Coleocentrus excitator* (Poda, 1761), female, hind coxa, trochanter and trochantellus.

**Figure 2e.**
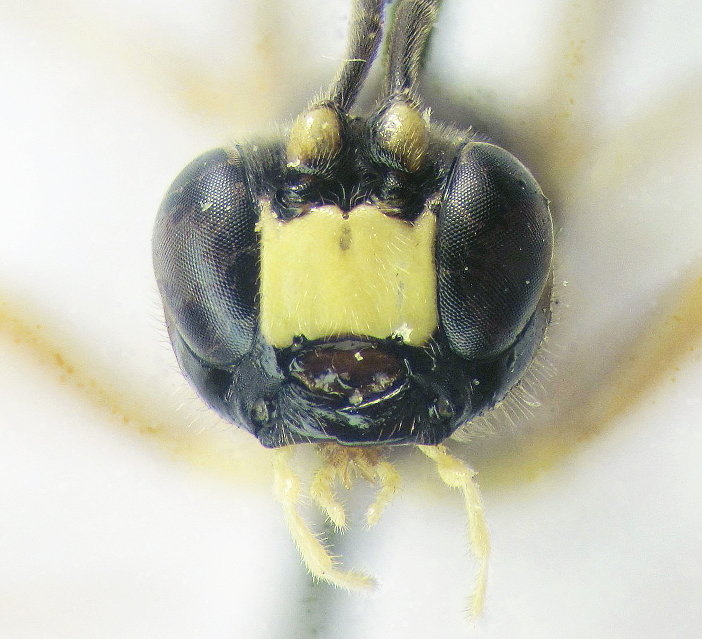
*Coleocentrus soleatus* (Gravenhorst, 1829), male, face.

**Figure 2f.**
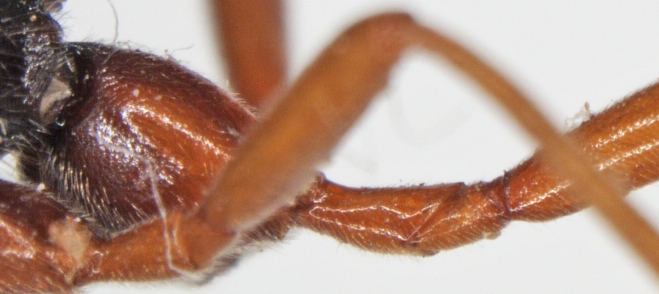
*Coleocentrus heteropus* Thomson, 1894, female, hind coxa, trochanter and trochantellus (holotype).
